# Stochastic attentions and context learning for person re-identification

**DOI:** 10.7717/peerj-cs.447

**Published:** 2021-05-05

**Authors:** Nazia Perwaiz, Muhammad Moazam Fraz, Muhammad Shahzad

**Affiliations:** School of Electrical Engineering and Computer Science, National University of Sciences and Technology (NUST), Islamabad, Pakistan

**Keywords:** Person re-identification, Attention, Context, Surveillance, Dropout, Deep features

## Abstract

The discriminative parts of people’s appearance play a significant role in their re-identification across non overlapping camera views. However, just focusing on the discriminative or attention regions without catering the contextual information does not always help. It is more important to learn the attention with reference to their spatial locations in context of the whole image. Current person re-identification (re-id) approaches either use separate modules or classifiers to learn both of these; the attention and its context, resulting in highly expensive person re-id solutions. In this work, instead of handling attentions and the context separately, we employ a unified attention and context mapping (ACM) block within the convolutional layers of network, without any additional computational resources overhead. The ACM block captures the attention regions as well as the relevant contextual information in a stochastic manner and enriches the final person representations for robust person re-identification. We evaluate the proposed method on 04 public benchmarks of person re-identification i.e., Market1501, DukeMTMC-Reid, CUHK03 and MSMT17 and find that the ACM block consistently improves the performance of person re-identification over the baseline networks.

## Introduction

**Figure 1:**
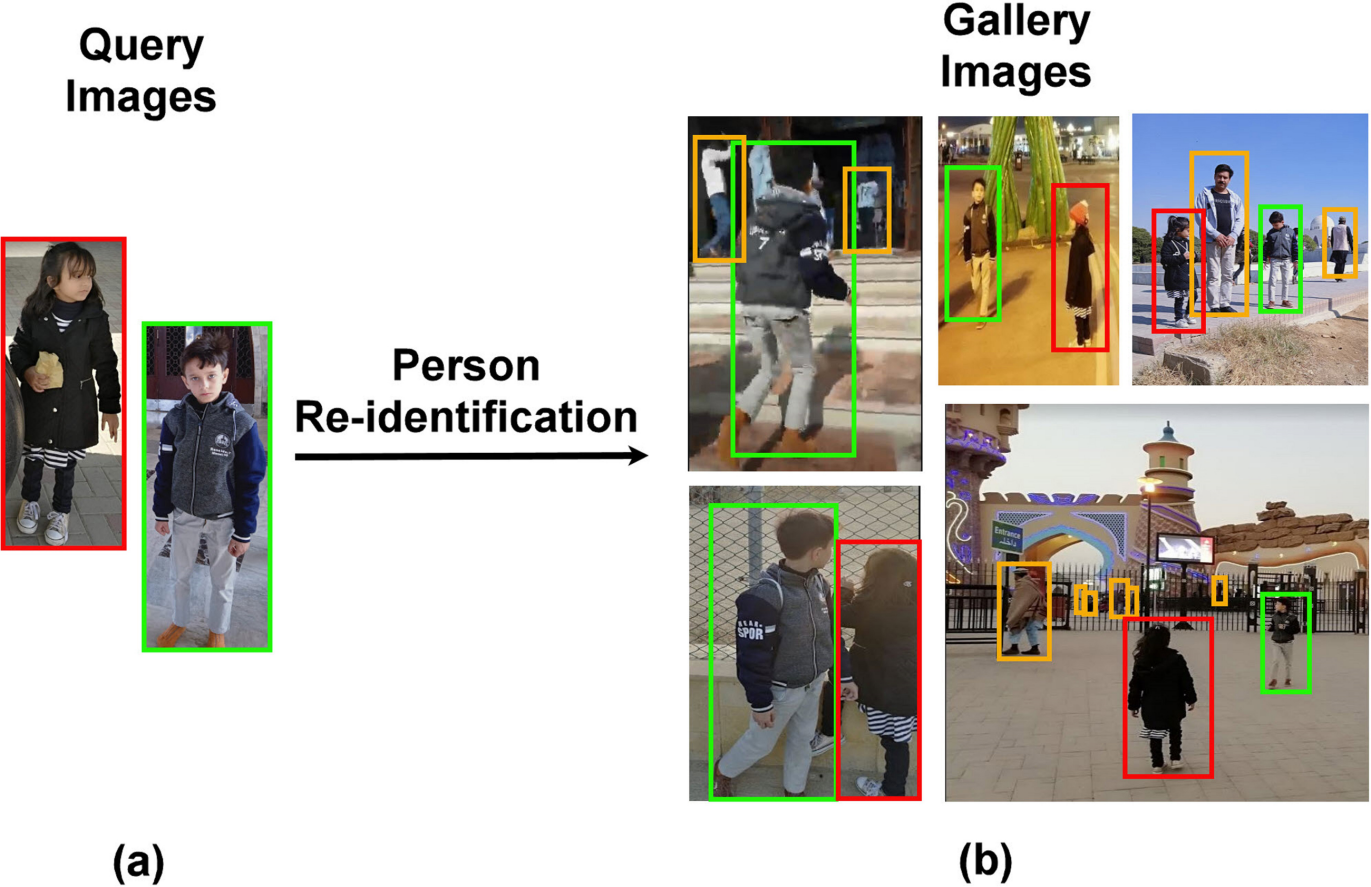
An intelligent person re-identification system identifies different people across multiple non-overlapping cameras of a surveillance network. (The color of bounding box illustrates same person re-identified in different cameras). (A) Query images (B) Gallery images.

Person re-identification (re-id) identifies a query person in multiple non overlapping camera views where the same person appears in different poses, angles and views under different illumination conditions. Person re-identification is one of the major surveillance components that needs automation to ensure 24/7 public security. Figure 1 illustrates an auto-surveillance scenario on the basis of intelligent person re-id system.

Generally, person re-identification is handled as a classification or retrieval task (Ansar et al., 2018; Batool et al., 2018). Each person with a unique id makes a separate class and all images of that person captured by different cameras belong to the same class. Since last few couple of years, various deep architectures are proposed to learn either global or local or both kinds of person representations (Zhao et al., 2017; Sun et al., 2019; Perwaiz, Fraz & Shahzad, 2020). Usually, the local salient features are learnt from predefined local parts, patches, strips etc and are then integrated with global person representations to perform person re-id.

Recently, with the success of attention based deep architectures in the field of computer vision (Zhang et al., 2018b; Woo et al., 2018; Mumtaz et al., 2017; mehdi Cherrat, Alaoui & Bouzahir, 2020; Zheng et al., 2019) the same are quickly opted for person re-identification domain as well (Li, Zhu & Gong, 2018; Liu et al., 2017; Perwaiz, Fraz & Shahzad, 2018; Mubariz et al., 2018). The attention based approaches extract local discriminative information by learning self-attention regions from the activation maps of convolutional layers instead of processing explicit predefined local parts of the image. These attention regions perform really well for various localization tasks (Dai et al., 2019; Kim et al., 2017), however need additional contextual information to tackle more complex tasks like person re-id which is challenged by huge intra-class variations. Few person re-id approaches integrate attentions and context via separate streams or classifiers for robust person representations, however such strategies increase the computational costs multi-fold (Si et al., 2018; Quan et al., 2019).

Other popular alternatives to learn the complete context erase random or specific parts of the image iteratively so that the model can be trained on all parts of image (Singh & Lee, 2017; Zhang et al., 2018a). Generally these methods partially turn off the activation map during the training phase to learn useful context from whole image. Random erase method randomly turns off different nodes at each hidden layer and focuses on the rest of image information to learn context information. Another random erase method (Zhong et al., 2020) erases comparatively bigger parts of the images instead of individual nodes, and learns useful context information from all parts of the images. These techniques are robust with no additional overhead on any baseline model and quickly train the model. Although these random drop based techniques improve the performances of Baseline models but more sophisticated ways to learn both the highly discriminative and less discriminative parts of the images are proposed by (Li & Xu, 2020; Liu et al., 2020; Perwaiz, Fraz & Shahzad, 2019). These multi-streams attention branches captures the discriminant attention features at different level but these approaches are quite expensive in terms of computations.

Although the attention based models are state-of-the-art but their need of high computational resources is a hurdle in designing large scale solutions. Meanwhile the need of attention based mechanisms, triggers the researchers to develop some light-weight attention based solutions for learning attention and context information in the images (Dai et al., 2019; Choe & Shim, 2019). In this work, we employ a mask based attention learning and dropping mechanism to learn the most discriminative parts of images along with the relevant context information from the image. Our contributions are summarized as follows:

1. We propose a light-weight attention and context learning (ACM) module to comprehend the higher level attention and context mappings, which does not require additional learnable parameters for self-attention computations.
2. We assimilate the ACM module within the baseline architecture as a plug-in component, hence the need of additional stream for self-attention learning is diminished.
3. The proposed method is computationally efficient and consistently outperforms the relevant architectures for four public person re-id benchmarks i.e Market1501, DukeMTMC-ReID, CUHK03 and MSMT17.

The next section describes related research work, in ‘Methodology’ the proposed methodology is discussed in detail. ‘Experiments’ provides the details of experimental materials and settings. The results and ablation studies are discussed in ‘Results and Discussion’ respectively. Conclusion and future research dimensions are discussed in the Conclusion and future work.

## Related Work

To prevent the over-fitting in deep learning models dropout (Srivastava et al., 2014) is a famous regularization technique. In a simple dropout method, the pixels of images are randomly turned off (turned to zero) in order to get their importance and impact on the training process. Generally, the neighboring pixels of an image relate to a single consolidated information, so the turning off of random pixels in hidden layers of a deep network does not make a sense of context learning in a bigger perspective.

In contrast to pixel wise dropout, (Zhong et al., 2020) analyzes the impact of dropping a small random patch from the images during the training. As in the images, a set of connected pixels contains some significant semantic information, the patch erase learning performs better than individual pixels level erase, but as the patches to be erased are randomly selected, it does not help a lot. Spatial dropout is another approach (Tompson et al., 2015) that avoids the pixel level dropout, chooses to drop a complete channel at a certain node spatial location, this also does not make sense as it rarely happens that discriminative information lies along the whole channel. The impact of simple random drop techniques open up the ways to choose the most effective dropout regions.

In this liu, (Park & Kwak, 2016) selects the maximum values of activation maps and turns them off, so that the model can learn from the rest of non-maximal activation nodes. This maximum drop method is applied both spatially and channel wise. However as it processes the pixel level values, it lack useful semantic information.

Recently, a batch wise dropout block (Dai et al., 2019) is proposed, which considers a complete batch of input images to drop a common region of activations along complete batch of the images, the dropped region selection is based on the attentive parts of the images instead of simple random drop. Attention based batch drop block uses a drop mask layer to achieve the goal with minimal computational resources, hence applicable at large scale but as it drops a fixed attentive region along complete batch images, there is a tradeoff for choosing the most attentive region along the whole batch of images.

Few techniques (Wang et al., 2017; Wang et al., 2018) explicitly capture the most discriminative parts of the images for the dropout purposes and perform really well for both localization and classification tasks. However these are computationally highly expensive and arise the need of some light-weight solutions.

In Choe & Shim (2019), Choe et al. propose a light-weight attention based dropout layer for weakly supervised localization tasks which detects the location of objects using image level labels instead of requiring explicit bounding boxes or masks in the ground truth. It computes the self-attention in each layer of a network and generates the importance and drop maps to learn both the attention regions and the context without requiring additional computational resources. Though this novel method performs well for localization tasks but it degrades the classification performance.

More specific to the dropout solutions for person re-identification, mostly the standard random dropout is used due to its inexpensive operations. The domain guided dropout (Xiao et al., 2016) and multi-branch architecture (Lin et al., 2019) learn the attention based person representations but these approaches are computationally very expensive. To the best of our knowledge, this work is the first one to learn the attention regions along with useful contextual information without needing additional computational resources, hence makes the proposed solution suitable for person re-id at the large scale.

In the proposed work, we employ an attention extraction procedure on the high level activation map. Instead of using the highly expensive explicit classifiers to compute attention regions, we choose to opt for attention based masks/ layers to get the high attention and less attention regions from high level image features. In contrast to the existing approaches which only concentrate on the discriminative parts of images, the proposed network learns the attentions as well as the relevant context with almost zero overhead on existing baseline network.

## Methodology

The proposed solution learns the highly discriminative regions of pedestrian images along with the non-discriminative regions which provide sufficient contextual information to recognize a person with negligible additional computational resources. The details about the baseline architecture, the proposed extension in the backbone network, formulation of the attention and context mapping module and the training pipeline are discussed in respective subsections.

### Baseline network

It is a common practice in computer vision research to use Resnet50 backbone network with Imagenet pre-trained weights, to design novel deep architectures (Hu, Shen & Sun, 2018; Roy, Navab & Wachinger, 2018). Following the same practice, we choose Resnet50 as the baseline architecture in the proposed work. From the high level features of the baseline network, we choose conv-4 output features (1) to generate the attention maps due to its wider spatial area than the final embedding of conv-5 layer. Moreover, we did not down-sample conv-4 layer embedding in order to generate attention and context masks from high resolution spatial activations. We split the baseline network in two splits, each of which carries the output embeddings of conv-4 layer. One split carries the original conv-4 features and the other one generates self-attention maps from the input embeddings to further highlight the discriminative and non-discriminative spatial regions within an Attention and Context Mapping block.

### Attention and Context Mapping (ACM) block

**Figure 2:**
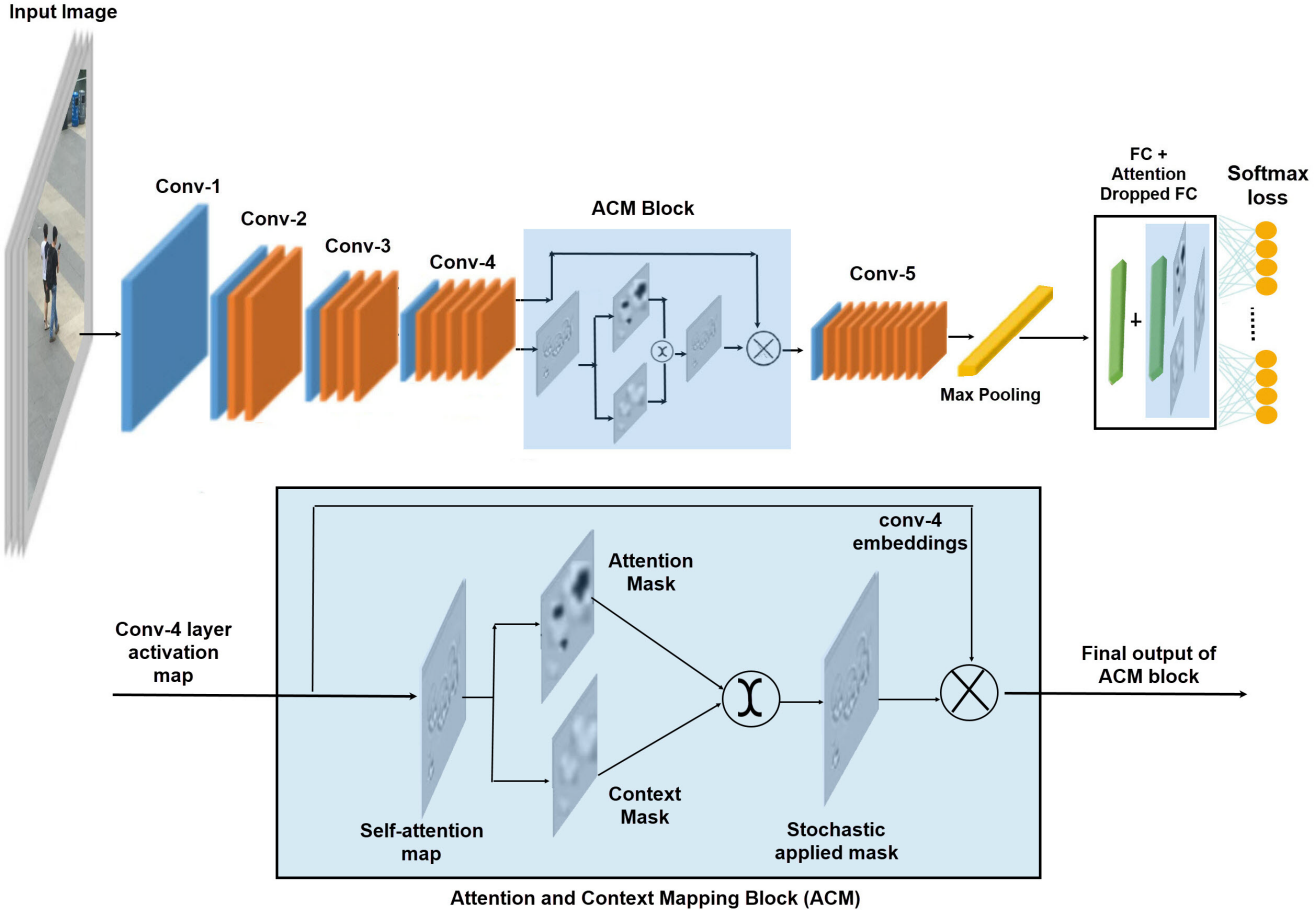
The proposed architecture; ACM block stack on to conv-4 layer and FC layer.

We achieve the attention and context mapping seamlessly, without any additional computational overhead by using light weight components. These light weight components are channel wise convolutions and the parameters free attention and context masks. We convert the set of conv-4 output feature map into the self-attention maps using light-weight channel-wise convolutions. The proposed ACM block is a two-stream structure, which receives the self-attention map to capture the contrast information from data. In one stream, it suppress the most attentive features by a simple function of threshold to create a drop mask, suppressing attentive features makes the model learn useful clues from the rest of contextual information. Instead of predefined drop patches, the size of region to be dropped is controlled by a hyper parameter and the attention drop rate. The other stream highlights attentive features by applying sigmoid activations on the self-attention map to create a mask of discriminative features. During the training process these masks are stochastically selected to highlight and learn the contrast information. The selected map is then element wise multiplied to the conv-4 layer embedding to enrich them before passing them to the final convolution layer of the baseline network. The whole process of ACM block is illustrated in Figure 2 and numerically explained in a sequential manner via (2) to (6):

(1) \begin{eqnarray*}Inpu{t}_{ACM}=Conv{4}_{OutFeatures}\subseteq {R}^{CXHXW}\end{eqnarray*}

(2) \begin{eqnarray*}SpatialAttentio{n}_{map}=ChannelwiseConv(Inpu{t}_{ACM})\subseteq {R}^{1XHXW}\end{eqnarray*}

(3) \begin{eqnarray*}Attentio{n}_{mask}=SpatialAttentio{n}_{map}\top Attentio{n}_{map}\end{eqnarray*}

(4) \begin{eqnarray*}Contex{t}_{mask}=SpatialAttentio{n}_{map} {null} Attentio{n}_{map}\end{eqnarray*}

(5) \begin{eqnarray*}Fina{l}_{mask}=Attentio{n}_{mask}{|}{|}Contex{t}_{mask}\end{eqnarray*}

(6) \begin{eqnarray*}Outpu{t}_{ACM}=Inpu{t}_{ACM}\otimes Fina{l}_{mask}\end{eqnarray*}

### ACM based fully connected layer

We obtain the final feature vector by applying global max pooling upon the final embedding of baseline network.

In addition to learning attention based rich person features, we introduce further sophistication in the learning of the classifier by applying similar attention and context learning maps on FC layer. We integrate the attentive FC layer with the standard FC layer (equation: (7)) before applying the loss function on it. We use cross entropy loss to train the proposed model.

(7) \begin{eqnarray*}F{C}_{final}=F{C}_{standard}+F{C}_{ACM}\end{eqnarray*}

### Training pipeline

Figure 2 illustrates the training pipeline of the proposed network, where person images are fed into the backbone network. A stack of convolutional layers augmented by residual connections learns the deep person representations. We plug-in the ACM block on top of the convolutional layer-4 of the base model, in order to apply the attention and context mapping on higher level feature map.

In the first phase, the ACM block generates the self-attention maps b y applying channel wise convolutions on the input feature map. Then, the self-attention masks are passed to the two complementary masks, one highlights the discriminative parts of the activation maps and the other erases the discriminative parts to focus on the context. These stochastic selection of the masks generates a final attention map, which is then applied to the original output features of conv-4 layer to embed the discriminative and contextual information stochastically. After incorporating the contextual information in final embedding of the network, max-pooling is applied to get the final embeddings. Additionally, we apply the attention-based dropping/ keeping of the nodes at the fully connected layer to embed the attention information in the classifier learning mechanism in a stochastic manner before applying the Softmax classification.

We do not employ the ACM block in the testing pipeline of proposed work, rather we use the standard conv-5 embedding to get feature vectors and compute Euclidean distance to find the similarity between given images.

## Experiments

### Datasets

We choose four public person re-id datasets to evaluate the proposed method which include Market1501 (Zheng et al., 2015); DukeMTMC-reID (Ristani et al., 2016); CUHK03 (Li et al., 2014)) and a recently proposed large scale MSMT17 (Wei et al., 2018).

**Market1501:** Market1501 is an image based person re-id benchmark having 1501 unique identities in it, out of which 751 identities are used for the training purpose and non-overlapping 750 identities are used in the evaluation. The dataset comprises 32,668 number of total person crops captured by 6 cameras, out of which 12,936 images make the training set, 19,732 images make the gallery and 3,368 images are used as query images.

**DukeMTMC-reID:** DukeMTMC-reID is an image based person re-id dataset and consists of 36,411 total number of pedestrian images captured by 8 different cameras. Training set covers 702 unique identities and the test set comprises 1110 unique identities, out of which 702 ids are used in the query set.

**CUHK03:** CUHK03 consists of 1467 unique identities with a total number of 14,097 images captured by 2 different cameras. 7365 images with 767 unique identities are used for the training purpose and the rest of 700 identities form the gallery set with a total number of 5332 images. 1400 person crops with 700 unique identities are used as query images. We use the newly defined and more challenging training/testing split protocol of CUHK03 dataset.

**MSMT17:** MSMT17 is a pretty large person re-id dataset. It contains 124,068 images captured by 15 cameras with 4101 unique identities in it. Out of 15, 12 cameras capture indoor images and 3 cameras capture outdoor images. The training set includes 1041 unique identities and 30248 image crops. The rest of 93,820 images with 3060 unique identities make the split of query and gallery set with 11,659 person crops of query images and 82,161 person crops in the gallery set.

### Evaluation metrics

For evaluation purposes, we choose following most commonly used metrics in the domain of person re-id research:

**Cumulative matching characteristics (CMC):** We use cumulative matching characteristics (CMC) for multiple ranks to compare our method with existing relevant approaches. The ranks of gallery images are computed on the basis of their similarity with query images.

**Mean average precision (mAP):** For multiple query images of the same person identity, the average precision of all query images is computed to find out the overall mean average precision of the system.

### Experimental settings

We use ResNet50 as our baseline network with ImageNet pretrained weight initialization. We use most commonly used data augmentation techniques horizontal flipping and random cropping in both the baseline and proposed architectures. The images are resized to 256x128 and the standard ImageNet normalization is applied to them. For optimization we choose Adam optimizer with the values of beta1 and beta2 0.9 and 0.999 respectively. The base learning rate is set to 0.0003 with the decay by a factor of 0.5 for every 20th epoch. We use the NVIDIA GPU model GeForce GTX 1080 Ti with the training batch size of 128 and testing batch size of 100 images. We train the baseline model, the proposed model and all of its variants for the maximum of 100 epoch for all datasets.

## Results

In the first phase, we use the baseline architecture i.e., slightly modified ResNet50 to train and evaluate it on all four person re-id datasets. And in the next phase, the impact of attention and context maps block is evaluated for all datasets. Re-id results as shown in Table 1, clearly illustrate that the inclusion of ACM block at feature learning level consistently performs better than the baseline model without ACM block.

**Table 1:** The comparison of proposed ACM based deep neural network with existing relevant person re-id approaches.

	**Market1501**		**DukeMTMC-ReID**		**CUHK03**		**MSMT17**	
**Method**	**mAP %**	**R1%**	**mAP %**	**R1%**	**mAP %**	**R1%**	**mAP %**	**R1%**
TriNet - RE [14]	69.14	84.92	53.5	72.44	**53.83**	**58.14**	–	–
SVDNet - RE [14]	62.1	82.3	56.8	76.7	37.8	40.9	–	–
IDE [30]	63.56	83.14	51.29	71.99	27.37	30.29	–	–
IDE + RE [14]	68.28	85.24	56.17	74.24	36.77	41.46	28.63	**59.77**
Baseline	68.3	84.4	58.2	76.8	44.3	47.5	25.8	49.4
Baseline + ACM block	**70.2**	**88.2**	**61.5**	**80.3**	47.3	53.6	**31.4**	59.7

**Note.**

The bold values indicate the best results.

**Figure 3:**
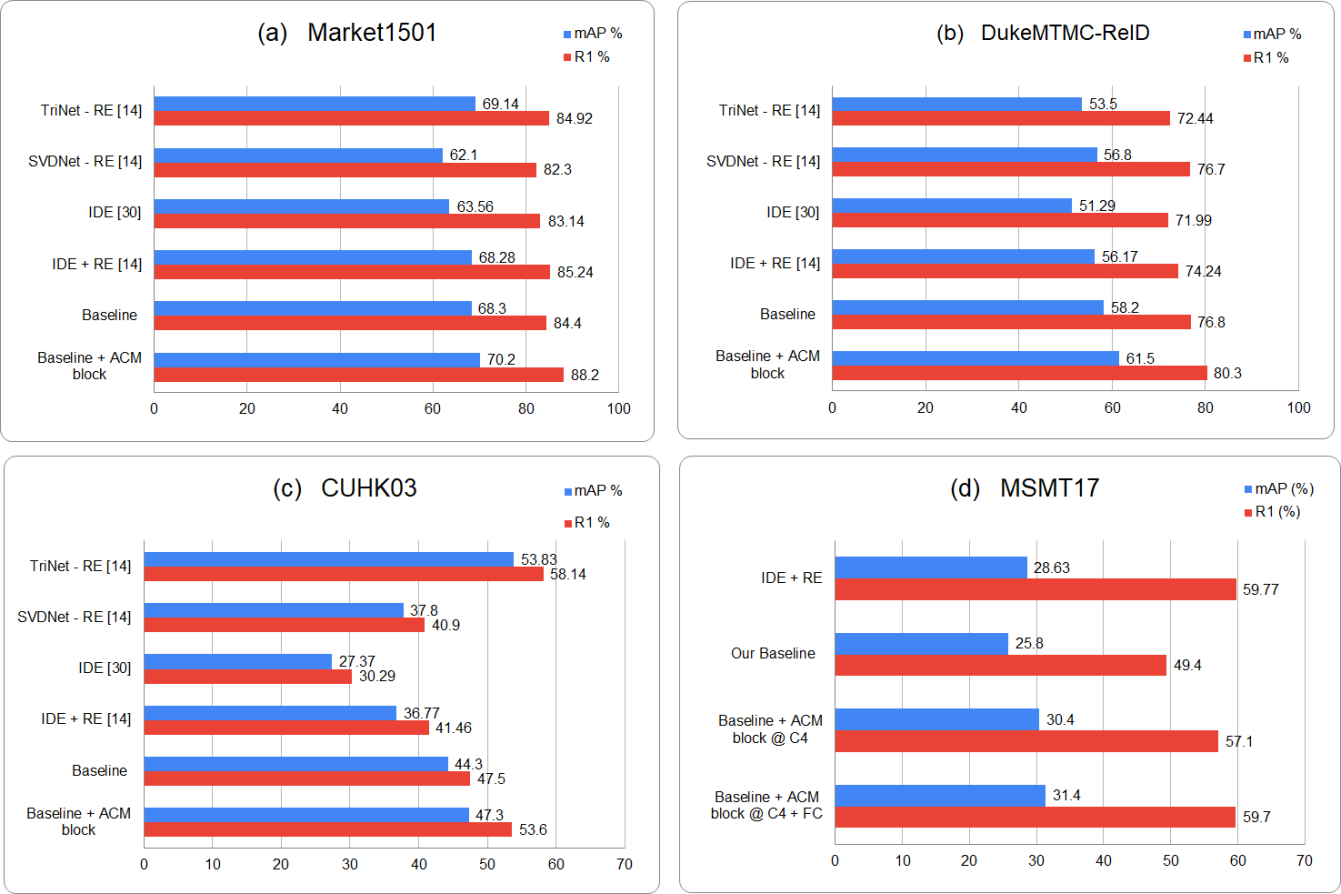
Graphical representation of the comparison of proposed results for four person reid datasets i.e., (A) Market1501, (B) DukeMTMC-ReID, (C) CUHK03 and (D) MSMT17, with the existing relevant Re-id approaches.

We also compare our results with existing relevant re-id methods. The results show that for the large scale re-id datasets -Market1501 and DukeMTMC-Reid, there is a significant increase in R1 accuracy and mAP over the rest of re-id methods. However, for CUHK03 dataset, which comprises far less number of images and unique identities, the triplet loss based trained model outperforms all of the classification based re-id methods including the proposed one. But the experiments still prove that the ACM block embedded model surpasses the baseline model with an increase of around 4% in R1 accuracy. Similarly for large scale re-id dataset MSMT17, ACM based model brings a huge performance improvement over baseline model and gets comparable results with other re-id methods. The graphical representation of results is available in Fig. 3.

**Figure 4:**
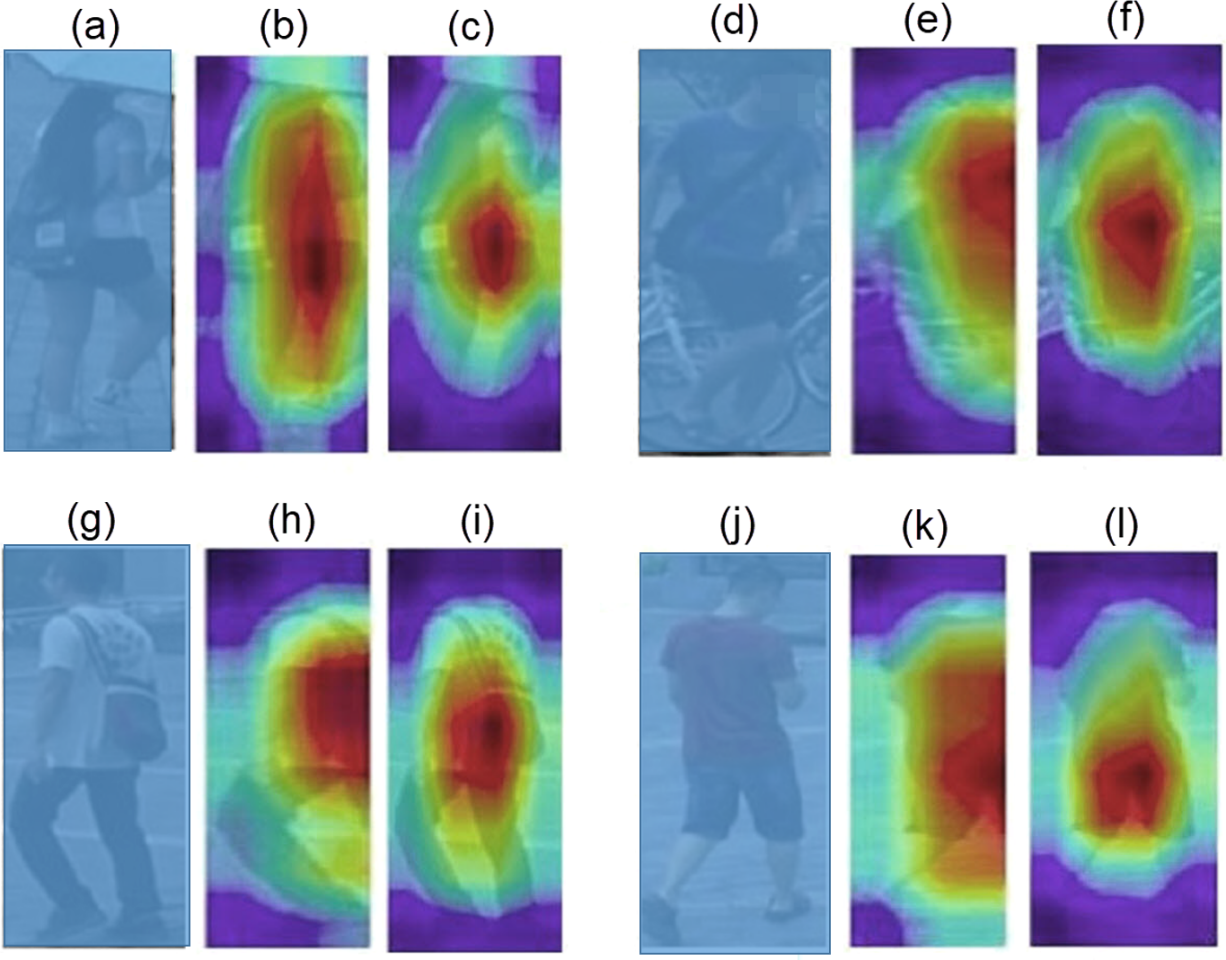
Visualization of Class Activation Maps: (A, D, G, H) The query images; (B, E, H, K) The class activation maps generated by the baseline model; (C, F, I, L). The class activation maps generated by the ACM integrated proposed model.

It is worth mentioning that ACM embedded models are trained in almost half the number of epochs than the baseline models. Almost all ACM based models converge around 20th epochs, whereas baseline models complete their training in more than 40 epochs. Figure 4 provides a qualitative comparison of class activation maps between the baseline and the ACM plugged proposed model. The more accurate highlights of the attention regions along with context information validate the significance of proposed network.

**Figure 5:**
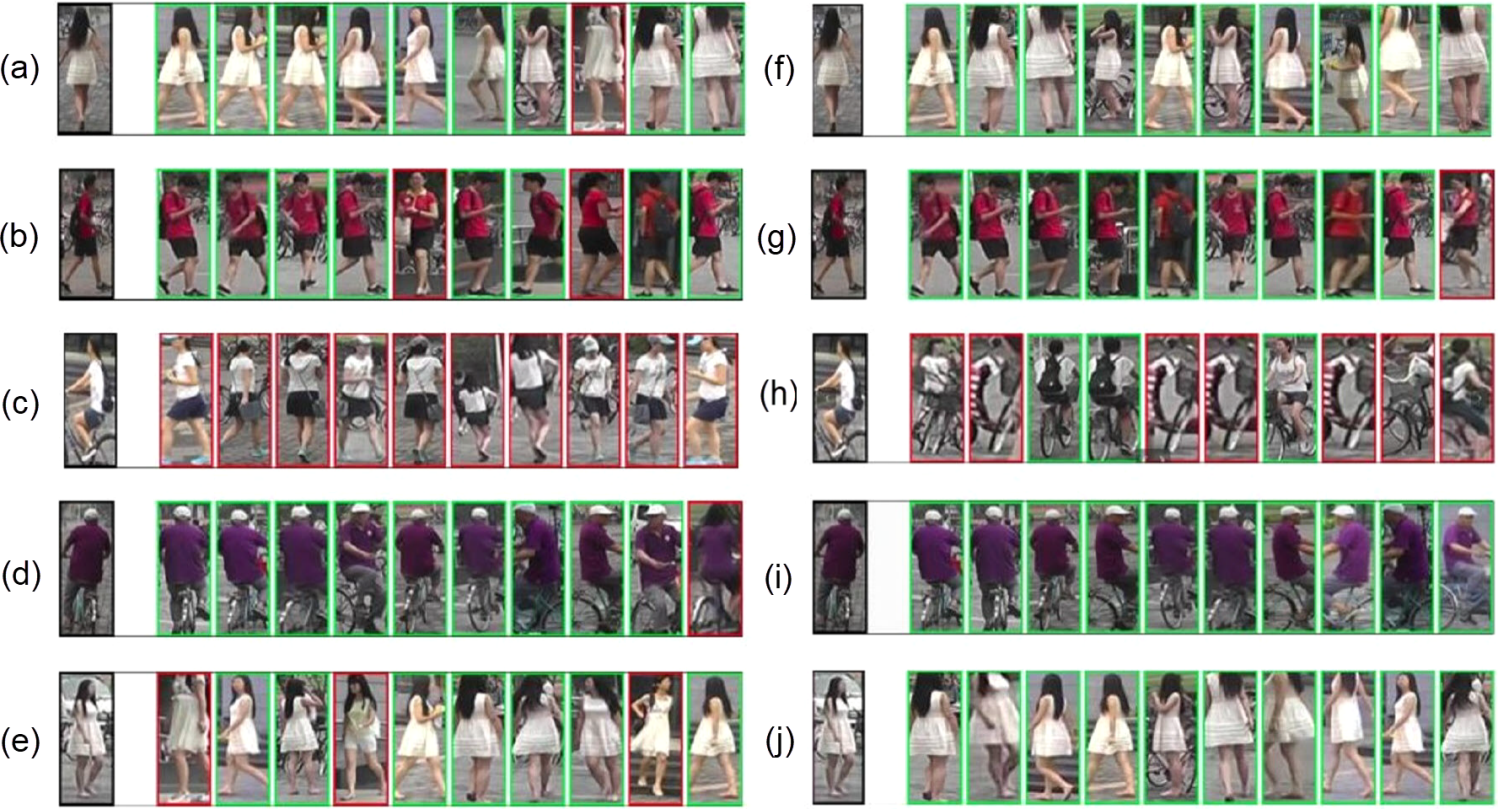
Qualitative results for top 10 close matches of given query images: (A–E) Results of baseline model with standard dropouts; (F–J) Results of proposed ACM based model.

Few query images from Market1501 dataset along with their top 10 predictions are given in Fig. 5. The ACM based model performs better than the baseline model, a difficult query image (as seen in third row of Fig. 5), that was not correctly identified even one time in the top 10 ranks by the baseline model, was accurately identified by the proposed method for three of its instances.

## Discussion

We conduct extensive experimentation on re-id datasets to analyze the impact of different components of the proposed solution and its variants. For each of the dataset, a comparative analysis is performed for the baseline model without any dropout settings, with standard dropout settings, with attention and context masks applied at all convolution blocks and with ACM blocks applied only at high level feature maps. The detailed results for each dataset are given in Table 2. Table 2A shows that employing ACM block only on high level embedding perform better than the baseline model and its standard dropout variants for Market1501 dataset. Further it is observed that for person re-identification, the low level attention drops do not play any significant role, rather it degrades the performance. However the learning and dropping of high level attention features in a stochastic manner improves the overall performance.

**Table 2:** The impact of standard dropout vs ACM based dropout variants on re-id accuracy and mean average precision for person re-id benchmarks (*LE =Layer-4 embedding).

**(A) Market-1501**						**(B) DukeMTMC-ReID**					
**Method**	**mAP %**	**R1%**	**R5%**	**R10%**	**R20%**	**Method**	**mAP %**	**R1%**	**R5%**	**R10%**	**R20%**
R50 + No dropout	68.3	84.4	93.5	95.8	97.4	R50 + No dropout	58.2	76.8	87	90.8	93.4
R50 + Standard dropout = 0.5	66.2	83.1	92.9	95.5	96.8	R50 + Standard dropout = 0.5	56.1	75.3	86.1	88.2	91.9
R50 + Standard dropout = 0.3	65.9	82.8	92.7	95.2	97	R50 + Standard dropout = 0.3	56.3	75.9	85.7	87.8	90.1
R50 + ACM @ All layers	61.4	81.7	92	94.9	96.6	R50 + ACM @ All layers	53.2	72.7	81.7	84.1	86.7
R50 + ACM @ LE	68.8	85.3	93.7	96.1	97.4	R50 + ACM @ LE	59.1	77.9	87.2	90.2	93.5
R50 + ACM @ LE & FC	**70.2**	**88.2**	**95**	**97**	**98.3**	R50 + ACM @ LE & FC	**61.5**	**80.3**	**88.4**	**91.2**	**94.8**
**(C) CUHK03**						**(D) MSMT17**					
R50 + No dropout	44.3	47.5	74.3	82.5	88.6	R50 + No dropout	26.8	52.7	69.5	79.3	82.8
R50 + Standard dropout = 0.5	42.4	43.2	61.7	71.9	80.7	R50 + Standard dropout = 0.5	25.1	48.6	67.1	73.9	79.7
R50 + Standard dropout = 0.3	42.8	43.4	63.4	72.6	80.7	R50 + Standard dropout = 0.3	25.8	49.4	67.8	74.4	80.6
R50 + ACM @ All layers	41.3	44.5	72.5	82.8	84.3	R50 + ACM @ All layers	23.7	50.8	64.7	75.9	78.6
R50 + ACM @ LE	45.5	51.2	75.4	82.4	88.9	R50 + ACM @ LE	30.4	57.1	71.8	77.9	83.1
R50 + ACM @ LE & FC	**47.3**	**53.6**	**76.4**	**84.6**	**91.1**	R50 + ACM @ LE & FC	**31.4**	**59.7**	**74.5**	**80.1**	**85.7**

**Note.**

The bold values indicate the best results.

For all four re-id datasets, we observe the consistent behaviour i.e., drop in the performances, for embedding ACM blocks at each convolutional level i.e., conv-1 to conv-5. In contrast, a consistent and significant improvement is observed for embedding ACM block at conv-4 self-attentions that represent high level attention features learnt during the training process. A comparative analysis for DukeMTMC-Reid is given in Table 2B.

Table 2C shows the results of CUHK03 dataset, we use the new training/testing split protocol for evaluation purposes, which turns out to be more challenging due to insufficient amount of images against each unique id. As we apply bare minimum modifications in vanilla settings of baseline model and loss function with no tricks and additional sophistications, the re-id performance on CUHK03 dataset does not meet the state-of-the-art results. However, the consistent behaviour of the proposed architecture over the baseline model clearly supports our claim about the significant impact of ACM block in learning person representations.

MSMT17 is a recently proposed large scale person re-id dataset with a huge size of gallery (i.e., 82161 images) and a pretty large query set (i.e., 11659 query images). The proposed architecture greatly improves the re-id performance over the baseline model and ensures the significance of the proposed ACM block. But as the MSMT17 dataset consists of a huge variety of images with different indoor and outdoor illumination and background settings, the overall re-id performances are not up to the mark as compared to the less complex re-id datasets Market1501 and DukeMTMC-Reid. MSMT17 results are given in the Table 2D.

**Figure 6:**
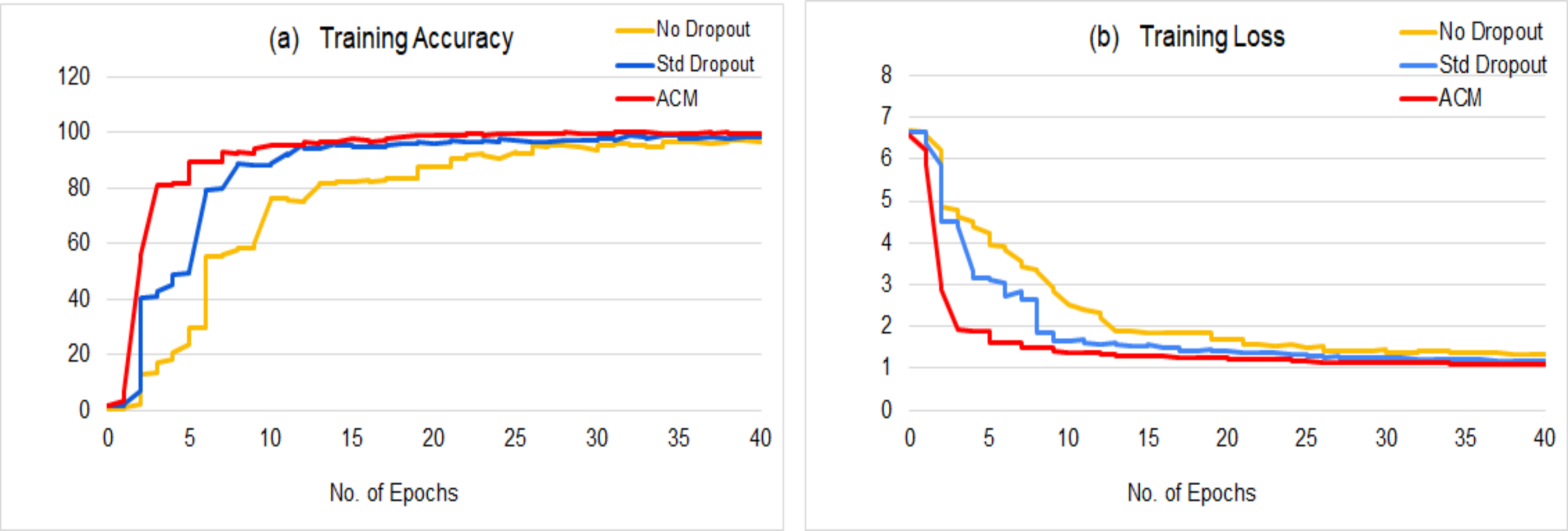
Comparison of the training statistics of proposed ACM based model with the baseline variants: (A) Training accuracy, (B) Training loss.

The proposed model reduces the training time to half of the training time taken by the base model, without increasing the number of learnable parameters and outperforms the baseline model with a great difference. Figure 6 shows a comparison between the training statistics of the proposed architecture and the baseline variants. The parameter-free ACM block boosts the network learning by emphasizing the attention and context without imposing any additional parametric overhead upon the network, and results in the quick convergence of the training process. ACM based model takes around 20 epochs to attain the significantly higher results (as reported in the Table 1), however the baseline model takes 40+ epochs to achieve its optimal results that are far lesser than the results of proposed model.

Moreover, in order to demonstrate the effectiveness of weight-less ACM module of the proposed network, we compare the computational complexity (in terms of trainable parameters) of the proposed method and a popular attention-based deep architecture i.e., Squeeze and Excitation network (SENet). The proposed ACM based re-id model attains even better results than the computationally expensive SENet with far-less number of trainable parameters. The computational efficiency of the proposed model is illustrated via Table 3. The results show that the proposed approach is parameter-free and equi-potential to the computationally expensive deep attention models.

**Table 3:** Computational efficiency of the proposed model in terms of network parameters.

**Method**	**Parameters**	**Flops**	**Epochs**	**mAP**	**R1**	**R5**	**R10**	**R20**
Baseline	23.498 M	2.568 G	40	68.3	84.4	93.5	95.8	97.4
SE-resnet50	26.039 M	2.520 G	25	69.8	87.2	95	96.7	98.1
Baseline + ACM block	23.498 M	2.568 G	20	70.2	88.2	95	97	98.3

## Conclusion and Future Work

In this paper, we propose a stochastic method to learn both the attention regions of images and the relevant contextual information using ACM block and without increasing computational overhead. This light-weight implementation involves the attention based masks to highlight or suppress the discriminative regions very efficiently. Extensive experimentation of the proposed method on 04 different public benchmarks of person re-identification - Market1501, DukeMTMC-Reid, CUHK03 and MSMT17 proves its consistent behaviour. It improves the performance of baseline architecture and reduces the training time of the model to half fold. Visual results of top close matches exhibit the learning capability of the proposed work on highly difficult data samples. In the future we plan to generalize the attention and context learning mechanism for cross datasets for practical implications of the research in real life surveillance systems.

##  Supplemental Information

10.7717/peerj-cs.447/supp-1 Supplemental Information 1 Python code.

## Additional Information and Declarations

### Data Availability

The following information was supplied regarding data availability: Code for this work is available at GitHub: https://git.io/Jt0vU
The DukeMTMC-reID dataset is available via GitHub: https://github.com/sxzrt/DukeMTMC-reID_evaluation). The procedure to obtained the DukeMTMC-ReID dataset is outlined in Github. Access to the CUHK03 dataset can be obtained by: (i) Clicking ’Download, and filling out the application form found at http://www.ee.cuhk.edu.hk/ xgwang/CUHK_identification.html. (ii) Please finish the form to obtain a download link. (This form is only to keep track of who owns the dataset.) Our code are available as a Supplementary File.
